# Spaceborne Synthetic Aperture Radar Survey of Subsidence in Hampton Roads, Virginia (USA)

**DOI:** 10.1038/s41598-017-15309-5

**Published:** 2017-11-07

**Authors:** D. P. S. Bekaert, B. D. Hamlington, B. Buzzanga, C. E. Jones

**Affiliations:** 1 Jet Propulsion Laboratory, California Institute of Technology, Pasadena, CA USA; 2 Center for Coastal Physical Oceanography, Old Dominion University, Norfolk, USA

## Abstract

Over the past century, the Hampton Roads area of the Chesapeake Bay region has experienced one of the highest rates of relative sea level rise on the Atlantic coast of the United States. This rate of relative sea level rise results from a combination of land subsidence, which has long been known to be present in the region, and rising seas associated with global warming on long timescales and exacerbated by shifts in ocean dynamics on shorter timescales. An understanding of the current-day magnitude of each component is needed to create accurate projections of future relative sea level rise upon which to base planning efforts. The objective of this study is to estimate the land component of relative sea level rise using interferometric synthetic aperture radar (InSAR) analysis applied to ALOS-1 synthetic aperture radar data acquired during 2007–2011 to generate high-spatial resolution (20–30 m) estimates of vertical land motion. Although these results are limited by the uncertainty associated with the small set of available historical SAR data, they highlight both localized rates of high subsidence and a significant spatial variability in subsidence, emphasizing the need for further measurement, which could be done with Sentinel-1 and NASA’s upcoming NISAR mission.

## Introduction

**Figure 1:**
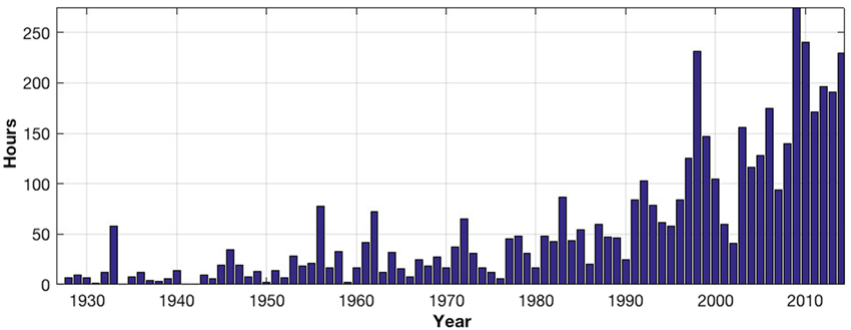
Hours per year of water-levels exceeding 30 cm above MHHW at the Sewell’s Point tide gauge.

The Hampton Roads area in the southern Chesapeake Bay region is experiencing one of the highest rates of relative sea level rise on the Atlantic coast of the United States due to a combination of land subsidence and naturally occurring (internal) ocean variability. Relative sea level refers to the movement of the ocean relative to land, with both components equally important when considering the coastal flooding. From the ocean side, several recent studies have identified a “hotspot” of sea level rise in the waters off of coastal Virginia, related to a combination of internal climate variability and global warming^1,2^. From the land side, Hampton Roads is undergoing subsidence at a rate only exceeded along the U.S. coastlines by locations on the Gulf Coast, particularly in areas where the rates in recent times are likely associated with or exacerbated by groundwater or oil/gas extraction^3,4^. The subsidence in Hampton Roads is assumed to be due to the presence of large-scale subsidence signals associated with glacial isostatic adjustment (GIA) and ongoing shifts associated with the Chesapeake Bay meteor impact crater that are known to impact the region^5^. This combination of ocean and land variability has led to a relative sea level trend of 4.6 + /− 0.2 mm/yr, as estimated from the Sewell’s Point tide gauge in Norfolk from 1927 to present^6^. The 20^th^ century rate of mean sea level rise is estimated to average 1.2 mm/yr to 1.9 mm/yr globally^7–9^, appears to be accelerating recently^10–12^, and shows considerable variability in sea level on the regional scale^13^. Associated with this high rate of relative sea level rise is a notable increase in coastal flooding over the last 90 years. Figure 1 show the number of hours per year of water levels exceeding a 30 cm threshold above Mean Higher High Water (MHHW) at the Sewell’s Point gauge. Between 1927 and 1990, the measured levels exceeded the 50 hr/year 7 times. After 1990, only one year with fewer than 50 hours/year can be observed, and with recent years experiencing greater than 200 hours/years.

Hampton Roads is home to the largest naval base (Naval Station Norfolk) in the world, and mitigating and adapating to future sea level rise is an issue of critical importance and national security. As such, planning efforts are already underway to address the sea level rise and increasing levels of coastal flooding in the region. Despite these efforts, there is still a lack of understanding regarding some of the contributors to the sea level problem in the region. In particular, information regarding the land contribution to the relative sea level rise is lacking. The most comprehensive subsidence measurements for the area cover the time period from 1940 to 1971, and although local rates of subsidence can be inferred from a combination of these historic measurements and sparsely located *in situ* observation (e.g., GPS), current subsidence estimates are insufficient – both in terms of accuracy and spatial resolution - for federal, state, regional and local governing bodies to develop locally precise adaptation and mitigation strategies to sea level rise^5^. In planning efforts, it has generally been assumed that subsidence across the region is relatively constant spatially and consistent with the rates measured from 1940 to 1971, varying only within a couple mm/yr over Hampton Roads. In part, this assumption is made because of the lack of higher-resolution information on vertical land motion.

While research is ongoing into the contribution of ocean variability to relative sea level rise, as discussed above, the land contribution (e.g., due to groundwater or oil/gas extraction) is lacking. Earlier studies utilizing GPS observations do not have the spatial resolution to capture the local horizontal land variability with an average station spacing larger than 20 km. This study seeks to resolve the gaps in our current state of knowledge and provide the first horizontal high-resolution (20–30 m) estimates of vertical land motion in the Hampton Roads region based on analysis of historic satellite SAR data using state-of-the-art time-series InSAR methods. Maps of the spatial variation of vertical land motion across the region with associated uncertainties are generated and used to identify the areas experiencing high rates of subsidence that will consequently be under the greatest threat from future relative sea level rise. The limitations of the historic SAR dataset over Hampton Roads are discussed, and suggestions made for future work that is necessary to improve knowledge of land motion in the region.

### Area of Study

**Figure 2:**
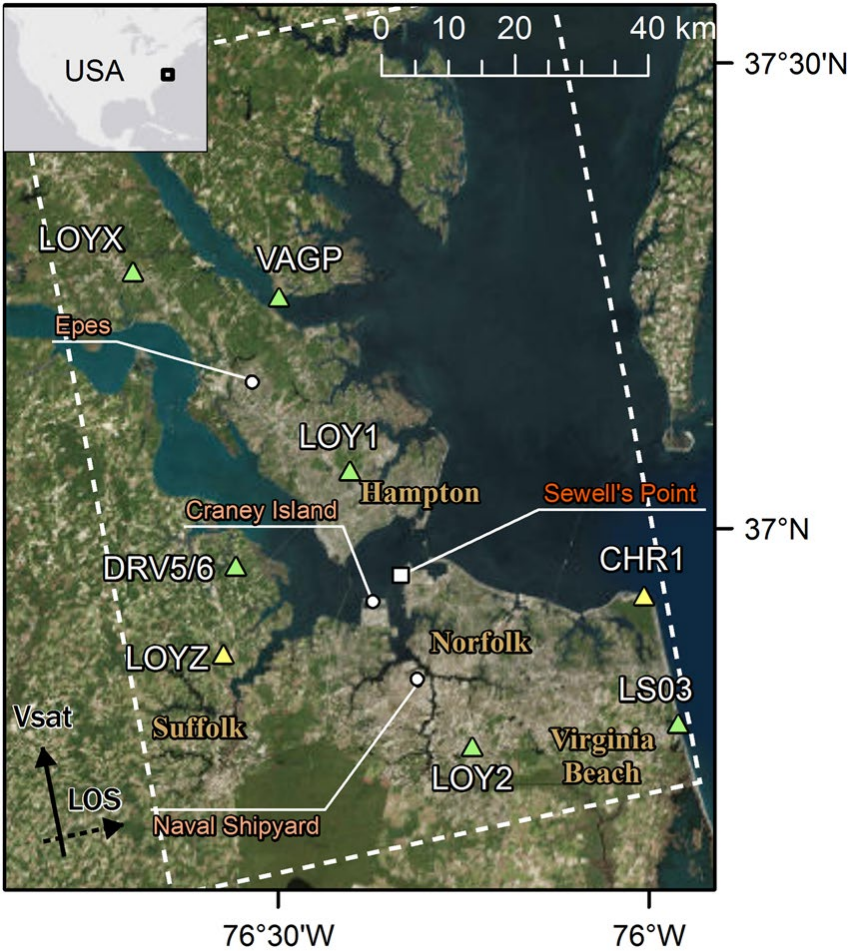
Area of study covering Hampton Roads, showing available GPS stations for reference. Green triangles represent stations used for the GPS reference frame tie-in, while yellow triangles represent stations used only for independent comparison. SRTM topography varies from -55 m tot 15 m. The ALOS-1 track used in this study is shown with the dashed line. ArcGIS 10.4 software (http://www.esri.com/software/arcgis) was used to generate the figure. Service layer credits ESRI, DigitalGlobe, GeoEye, Earthstar Geographics, CNES/Airbus DS, USDA, USGS, AeroGRID, IGN, and the GIS User community.

The area of study is the Hampton Roads region of southeastern Virginia (Fig. 2), which contains a mixture of urban areas, agricultural land, and coastal wetlands. Interferometric Synthetic Aperture Radar (InSAR) has proven to be a powerful technique to study earth surface displacements^14^. Measurement of subsidence using InSAR in agricultural areas is notoriously difficult because of temporal decorrelation, which causes loss of signal coherence over time^15^. It is much easier to measure subsidence in urban settings because hard targets provide consistent, coherent targets that can be processed with time series InSAR methods, which have been used successfully in similar settings to the Hampton Roads area and with similar required accuracy (1–4 mm/yr)^16–19^. Although the larger goal of this work is to quantify subsidence and sea level rise rates throughout the Hampton Roads area, both urban and agricultural, the primary focus here is on determining local subsidence rates in the populous areas and at the facilities in Norfolk, Virginia Beach, Portsmouth, Hampton and Newport News that are critical to national security, particularly ship yards, military installations, power facilities, and water treatment facilities.

### Data

**Figure 3:**
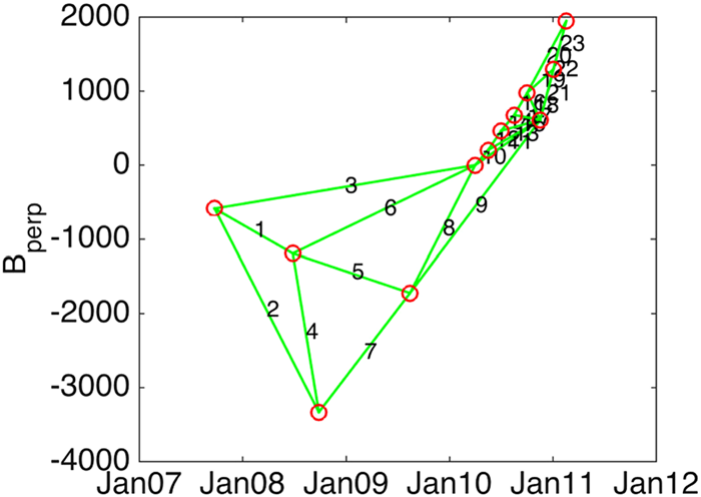
ALOS-1 small-baseline plot showing the perpendicular baseline for the 12 acquisitions (red circles) of the time series. Green lines indicate the interferograms that were used in the time-series analysis.

This study used available historic satellite SAR data acquired from the Japanese ALOS-1 satellite between 2007 and 2011. This data is freely available through the Alaska Satellite Facility. Figures 2 and 3 show the available ALOS-1 data acquired in FBD or FBS beam modes and the number of acquisitions during the period of study (2007–2011). A minimum of 10–12 acquisitions is recommended for time-series processing^14^, and met for the study area by the two scenes bisecting Norfolk, VA, on track 137 (highlighted in Fig. 2). These two scenes cover a range of land types, both urban and vegetated, allowing for a determination of the suitability of the applied analysis across the entirety of Hampton Roads. Figure 3 shows the distribution of the 12 acquisitions across the time series, and shows a notable increase in acquisition frequency during 2010 and 2011. The considered interferogram pairs along with relevant baselines are also shown in Fig. 3 and summarized in Table 1. Interferograms 2, 4, 7, 9, 19, 21, and 23 were removed from the processing due to large baselines and the presence of large ionospheric path delay errors.

**Table 1:** Baseline information of the interferograms used in the small baseline time-series InSAR processing.

Interferogram	Time Period	Days Covered	Baseline (m)
1	20070924–20080626	276	−604
2	20070924–20080926	368	−2745
3	20070924–20100401	920	586
4	20080626–20080926	92	−2140
5	20080626–20090814	414	−532
6	20080626–20100401	644	1190
7	20080926–20090814	322	1608
8	20090814–20100401	230	1723
9	20090814–201117	460	2333
10	20100401–20100517	46	204
11	20100401–20101117	230	611
12	20100517–20100702	46	261
13	20100517–20101117	184	406
14	20100702–20100817	46	213
15	20100702–20101117	138	146
16	20100817–20101002	46	300
17	20100817–20101117	92	−67
18	20101002–20101117	46	−367
19	20101002–20110102	92	319
20	20101002–20110217	138	971
21	20101117–20110102	46	686
22	20101117–20110217	92	1338
23	20110102–20110217	46	652

Interferograms 2, 4, 7, 9, 19, 21 and 23 were dropped during processing (see text for more information).

The relative subsidence map derived from InSAR is combined with GPS rates (NA12 plate reference) generated at the University of Reno^20^ in order to tie the InSAR rates to an absolute GPS reference frame. We performed a visual inspection of individual GPS time-series using the online portal (http://geodesy.unr.edu/) to make sure the rates can be assumed stable in time and to also confirm the automated system captured any cabeling and antenna changes correctly. In the area of study, there are eight available GPS stations (Fig. 2). The measured vertical rates and associated time periods for each GPS station are shown in Table 2. Six of the GPS stations were used to tie the InSAR rates with the reference frame. One of the stations provided measurements that did not overlap our study time period, and another was located in an area with relatively few valid InSAR measurements. Both of these GPS stations were omitted from the process of transferring the InSAR rates into an absolute GPS reference frame, but were used as independent points of comparison.

**Table 2:** Station name and calculated horizontal and vertical linear GPS rates with corresponding 1-sigma uncertainties for the vertical rate.

Name	East Vel. (mm/yr)	North Vel. (mm/yr)	Vert. Vel. (mm/yr)	Uncert. Vert. Vel (mm/yr)	Time Period	Used as Ref.
LOYX	−0.4	0.01	−1.89	0.93	20090509–20170408	Yes
VAGP	−0.4	0.01	−2.24	1.21	20071001–20160419	Yes
LOY1	−0.22	0.06	−2.42	1.45	20090206–20120321	Yes
DRV5 DRV6	−0.32	0.07	−1.86	0.48	20060310–20160818	Yes
LOYZ	−0.14	0.36	−1.80	0.82	20090220–20170408	No
LOY2	−0.28	−0.01	−1.77	0.86	20090206–20170408	Yes
LSO3	−0.14	−0.13	−0.98	0.90	20090255–20170408	Yes
CHR1	−0.14	−0.62	1.40	1.53	19960114–19990618	No

The time period of each GPS record is shown in addition to whether it was used in the reference frame correction. For stations within a 250 m radius a weighted average rate and uncertainty was calculated.

### Vertical Rate Estimates for Hampton Roads

**Figure 4:**
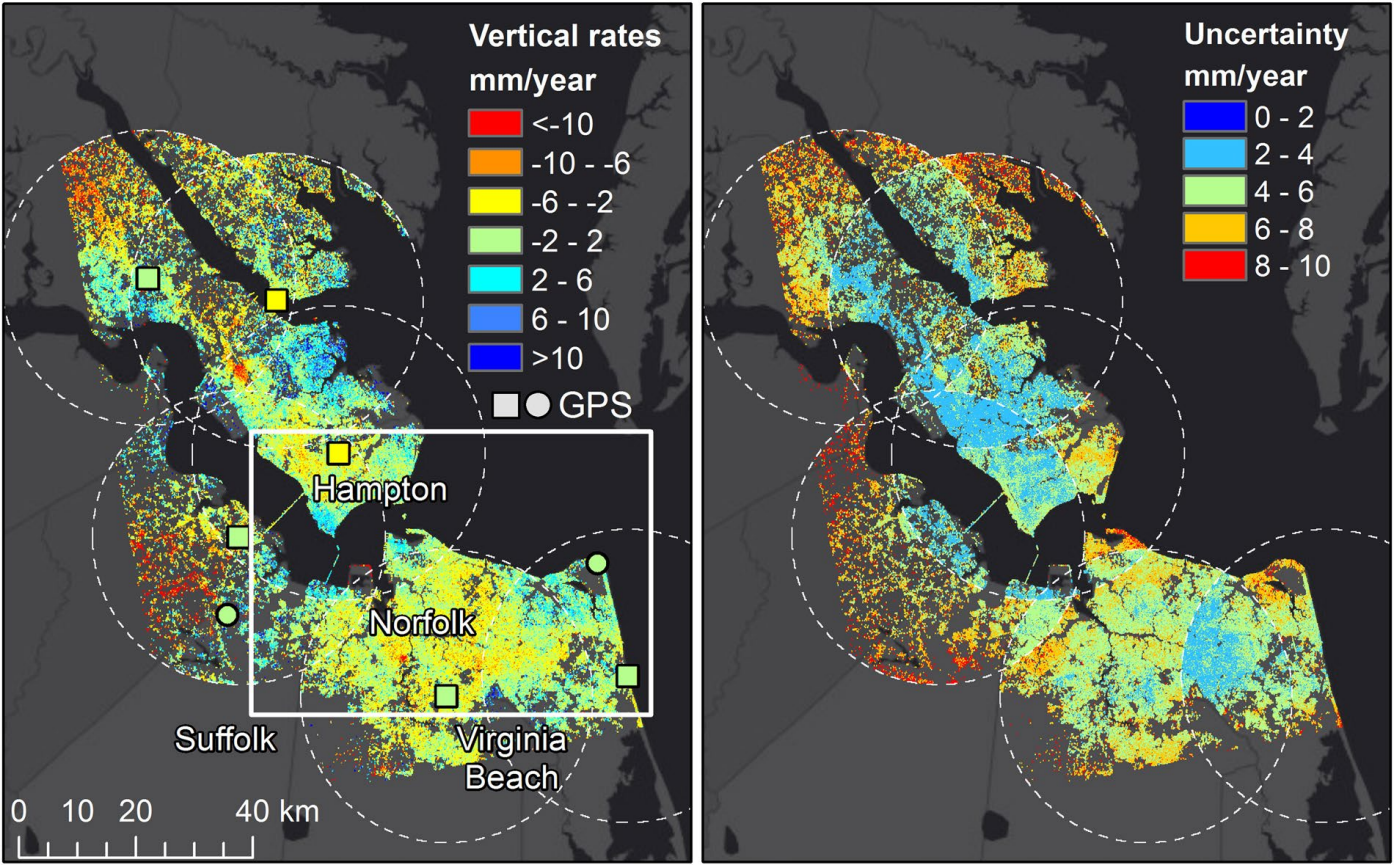
Subsidence rate-map (left) and corresponding uncertainties (right) as estimated from historic (Sept 2007-Feb 2011) ALOS SAR data referenced to a GPS reference frame. Left, due to longer wavelength spatial variation (>20 km) of ionospheric InSAR noise, only InSAR subsidence rates within a 20 km radius of a GPS site (dashed circles) are use to locally reference InSAR to the vertical GPS rate. A weighed average is performed in the circle overlap regions to integrate the local referenced InSAR subsidence in a regional map. Right, propagated uncertainties shows the uncertainty grows with distance from a GPS station due to atmospheric noise, and areas falling within multiple circles to have a smaller uncertainty due to noise averaging. GPS-measured rates are shown in square and circular markers on the subsidence rate map, with the square markers representing the stations used for the GPS referencing and the circular markers stations used for comparison only. ArcGIS 10.4 software (http://www.esri.com/software/arcgis) was used to generate the figure. Service layer credits ESRI, HERE, DeLorme, MapmyIndia,^©^ OpenStreetMap contributors and the GIS User community.

After processing the ALOS-1 data using the methods discussed below, a vertical rate map for Hampton Roads over the time period from 2007 to 2011 is obtained (Fig. 4, left) along with the associated uncertainty in the rate (Fig. 4, right). The available GPS-measured rates are also shown for comparison (square markers are used for GPS stations used to tie into GPS NA12 plate reference frame), showing good agreement with the underlying InSAR-generated rate map. Due to long-wavelength errors introduced primarily due to atmospheric (ionosphere and troposphere) noise in the InSAR data^14^, we combine the two datasets using the InSAR data to constrain the short spatial scales and the GPS data to constrain subsidence across longer spatial scales. Specifically, the vertical InSAR rate and uncertainty maps were estimated with respect to each individual GPS reference station, using only InSAR data within 20 km of a GPS station (Fig. 4, circles), thereby removing any long-wavelength signal. We then computed a weighted average of the InSAR data, using the estimated rate uncertainty for the weighting. After propagating the uncertainties accordingly, the rate and uncertainty maps shown in Fig. 4 are produced. As a result of this procedure, locations near to a GPS station have reduced uncertainty when compared to areas without a GPS station nearby (see methods for more details).

Although in many areas the uncertainty is large and the estimated rates are not significant at either the one- or two-sigma levels, there is still important information that can be drawn. From a broad perspective, significant spatial variability in rates of vertical land motion exists around the region. Contrary to commonly thought, there are a number of areas experiencing uplift over the time period, particularly in the vicinity of Poquoson and York County (north of LOY1), and in Cape Henry (near CHR1). Further focusing on the area of low uncertainty around Hampton and Newport News, there are relatively large changes in vertical land motion over short spatial scales. Over a roughly 20 km range extending west to east through Hampton, there is a shift from subsidence to uplift. Along the eastern shore in Virginia Beach, a gradual change from uplift to subsidence (north to south) in observed, with these rates confirmed by two available GPS stations in the vicinity.

This apparent spatial variability in the vertical rates likely precludes a “one size fits all” approach to planning for subsidence in the region. The associated uncertainty estimates (Fig. 4, right) also demonstrate the importance of *in situ* observations in the form of GPS measurements. InSAR-measured rates in the vicinity of a GPS station have significantly lower uncertainty than those further afield of GPS. This is particularly notable for northern Norfolk, which has rate uncertainties on the order of 1 cm/yr and few GPS stations.

**Figure 5:**
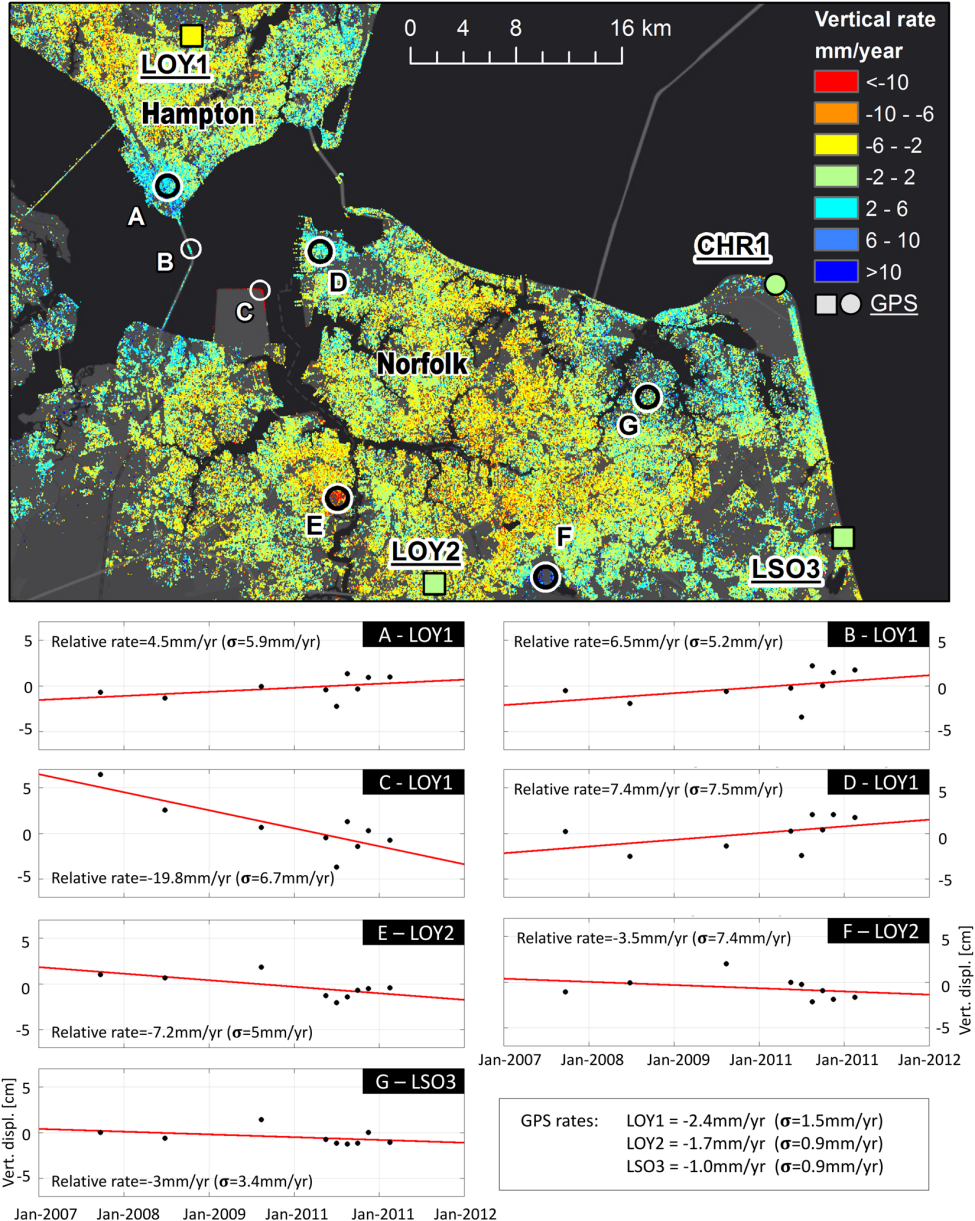
Profiles drawn across the subsidence map (left) showing change in rates along each transect (right). Uncertainty is shown across each profile, with dark gray showing the one-sigma uncertainty, and lighter gray representing the two-sigma level. Each measurement along the profile is obtained by including the InSAR rates within a 500 m radius about the point along the profile. Rates measured at the GPS stations that are found along the profiles are shown in red (if used in inversion) and light-blue with 2-sigma uncertainties, and labeled with the station name. Matlab R2015B (https://www.mathworks.com/) and ArcGIS 10.4 software (http://www.esri.com/software/arcgis) was used to generate the figure. Service layer credits ESRI, HERE, DeLorme, MapmyIndia, © OpenStreetMap contributors and the GIS User community.

As an additional method of viewing the spatial variability of vertical land motion and uncertainty in the region, three profiles are drawn, transecting the region of study (Fig. 5, left). Profile A-A’ begins on the eastern shore of Virginia Beach and ends in the northwest portion of the region. The large uncertainty for much of the region is clear (indicated by the gray-shaded error bars), but a number of features stand out with exceptional rates of vertical land motion including subsidence signals in the Norfolk Naval Shipyard and Craney Island land reclamation project. To the north (end of A-A’ profile), further subsidence is found, including near the closed landfill in Epes, Newport News. The rates at the GPS stations along the profile are shown in red and green and demonstrate good agreement with the underlying InSAR map. Two further profiles are drawn, one from Cape Henry to Chesapeake/Suffolk (B-B’) showing a transition from an area of uplift to an area of subsidence, and one starting from Smithfield/Isle of Wight (C-C’) and showing agreement with several of the available GPS stations in the area.

### Time Series Analysis

**Figure 6:**
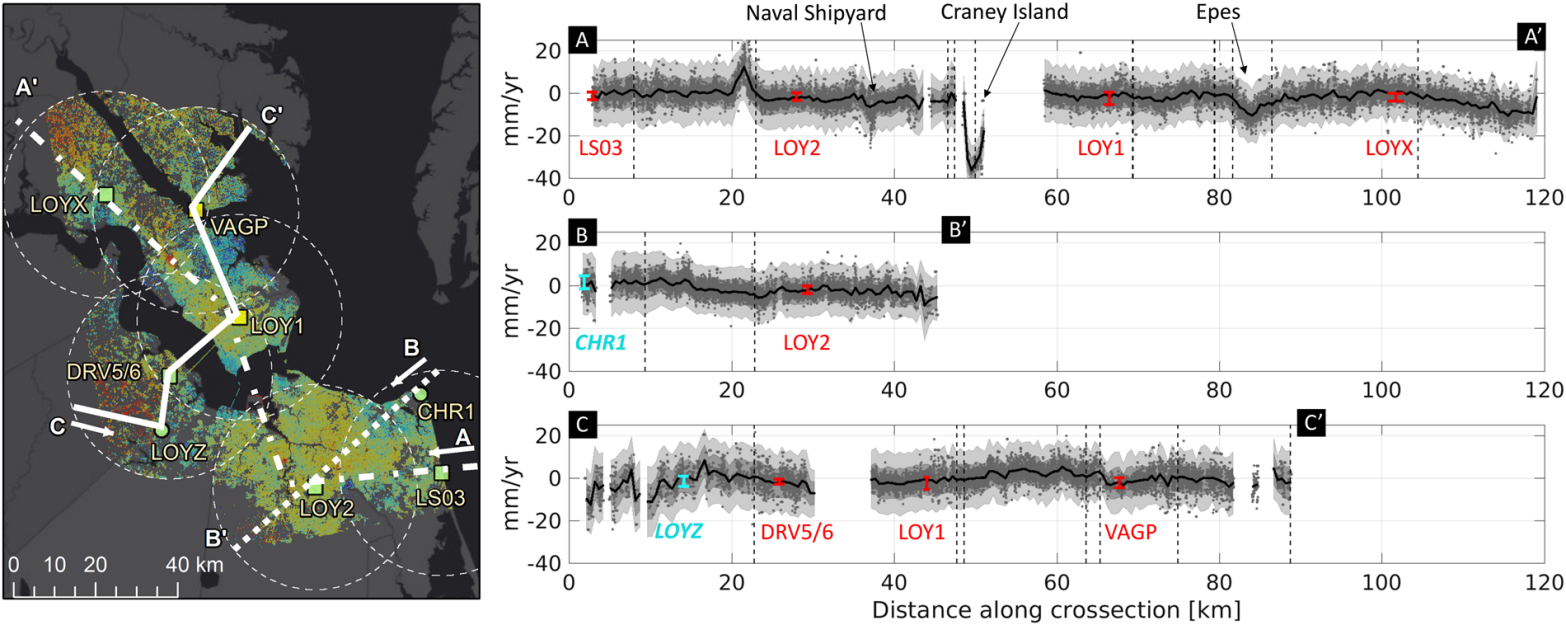
Vertical rate map focused on the area centered on Norfolk (box shown in Fig. 2A). Bottom panels show differenced time series for seven locations relative to the nearest GPS station. Matlab R2015B (https://www.mathworks.com/) and ArcGIS 10.4 software (http://www.esri.com/software/arcgis) was used to generate the figure. Service layer credits ESRI, HERE, DeLorme, MapmyIndia,^©^ OpenStreetMap contributors and the GIS User community.

Two primary benefits of the approach used here are the representation of subsidence on small spatial scales and the ability to analyze the time series of vertical land motion at each location. In order to identify areas that are experiencing large rates of subsidence, we focus on a region centered on Norfolk and encompassing Hampton, Virignia Beach and Hampton (among other areas) (box shown in Fig. 4A, zoomed in area shown in Fig. 6, top). Differencing the time series of two nearby locations has the benefit of reducing the atmospheric error, which generally varies over longer spatial scales. Here, we select several points and difference the InSAR measured time series relative to the InSAR measurements surrounding a nearby GPS station. It should be noted that this difference is built upon the assumptions that the respective time series are linear and that the InSAR observations near the GPS stations are accurate and agree with the GPS measured rates.

Seven separate points are selected for time series analysis: (A) southern Newport News, (B) Island where Monitor Merrimack bridge connect to a tunnel, (C) Craney Island, (D) Old Dominion University in Norfolk, (E) Norfolk Naval Shipyard, (F) southeast Virginia Beach, and (G) the Kings Grant area of Virginia Beach. The time series for each point relative to the nearest GPS station are shown in the bottom panels of Fig. 6. Significant rates of subsidence exceeding the two-sigma level are found at Craney Island, and exceeding the one-sigma level at the Naval Shipyard, while a significate rate of uplift is found on the bridge (one-sigma), consistent pattern with a time-series InSAR study over the bridge using RADARSAT-1 data^21^. During the study time period (2007–2011), Craney Island was expanded eastward through a land reclamation project. This expansion is being accomplished through the disposal of dredged material that is then contained by the surrounding dikes. This additional loading appears to be causing subsidence in the dikes that is occurring at a higher rate than the addition of dredged material, which has been monitored with *in-situ* measurements. The cause of the subsidence at the Norfolk Naval Shipyard is unknown. Although the other selected locations do not individually show statistically significant rates given the uncertainty, the observed rates do highlight the spatial variability of vertical land motion across the area of study.

## Discussion

As discussed above, the Hampton Roads coastlines are experiencing exceptionally high rates of relative sea level rise, owing to both the steady increase of sea level and the long-term subsidence of the land. The rate of global mean sea level rise over the past century is approximately 1.5 mm/yr. On the other hand, the relative average sea level rise measured at Sewell’s Point gauge in the Hampton Roads region from the 1950 through the present is on the order of 4 mm/yr. While some of this increased rate could be ocean-related, these first order data imply a significant level of subsidence in the region. Specific – albeit sparse – previous measurements of subsidence, indicate a broad range of subsidence that could be even greater in some localities. Subsidence measurements based on geodetic surveys made from 1940 to 1971 indicated an average subsidence rate of 2.2 mm/yr with a range of 1.1 to 4.8 mm/yr across the region^22^. These data were updated with GPS CORS measurements taken between 2006 and 2011 that showed an average subsidence rate of 3.1 mm/yr at three GPS stations (VAGP, DRV6 and VIMS) in the region^23^. These stations are shown in Fig. 2, except for VIMS which is outside our study area. The stations, inland at Franklin, Virginia, and closer to the Hampton Roads littoral at Suffolk, Virginia, showed subsidence rates of 1.5 and 3.7 mm/yr respectively, based on groundwater extensometer measurements taken between 1979 and 1995^24^. While the Suffolk station is within the ALOS frame boundary, there are no collocated InSAR measurements. In short, there is evidence of high rates of subsidence that appear to vary considerably across the region.

To date, little has been done to improve upon this spatially sparse information and satellite observations have been a heretofore-untapped resource. The results presented here represent a first attempt at filling a gap in current knowledge regarding the threat of future sea level rise that is needed for planning and mitigation efforts in the Hampton Roads region. While the available historic ALOS SAR data is limited (Fig. 4), there are clear indications of spatial variability in vertical land motion with some areas experiencing high rates of local subsidence. Given the costly infrastructure in Hampton Roads, identifying these areas has important implications for future planning efforts. From this initial analysis, for example, Craney Island and the Norfolk Naval Shipyard appear to be experiencing high rates of subsidence. While the subsidence at Craney Island is perhaps unsurprising given the additional loading the area experiences on a regular basis, there is at present little explanation for the apparent subsidence at the Naval Shipyard from 2007 to 2011. If this rate has continued on past 2011 and indeed continues on into the future, the Naval Shipyard would be expected to be ever increasing threat for future sea level rise.

There are a number of issues with the historical SAR data and the results presented here that limit their implementation as the basis for planning efforts. First, the estimated rates reflect only the land motion from 2007 to 2011, which is assumed to be linear. Whether or not these can be extrapolated into the future depends on the causes of the vertical rates and whether land use remains consistent. In addition, uncertainties would also scale over time. If loading in Craney Island were to cease, for example, there would be an expected associated change in the vertical rate. Second, the observations provided by the ALOS-1 satellite limit the quality of the vertical rate information that can be extracted. The L-band measurements are particularly sensitive to the ionosphere, leading to increased uncertainty from that source of atmospheric noise^25^. The number and intermittent nature of the available acquisitions are also less than ideal for estimating linear vertical rates^14^. These inherent challenges underscore the need for further analysis. Given the thermal and scattering noise of the sensor in combination with the data sampling and atmospheric noise we find at best an uncertainty of 3–4 mm/yr for the derived rates.

Since 2015, the Sentinel-1 satellite has been acquiring data over the Hampton Roads region. Starting in September, 2016, Sentinel-1 began acquiring data over Hampton Roads every 12 days. Additionally, the Sentinel-1 satellite samples in the C-band, leading to a dramatic reduction in the uncertainty associated with the ionosphere. Importantly, the European Union Commission has committed to continuing and augmenting the Sentinel constellation until at least 2030, ensuring the ability to monitor subsidence over Hampton Roads and leading to dramatically reduced uncertainties as the time series gets longer. In summary, while analysis of the Sentinel-1 data should eventually provide the decision-making quality vertical land motion maps that are needed for Hampton Roads, the results presented here motivate and demonstrate the need to improve the understanding of the vertical land motion in the region.

## Methods

Interferometric Synthetic Aperture Radar (InSAR) has proven to be an attractive technique to provide high-spatial resolution (up to few meter) observations of surface displacements, assuming a coherent signal is maintained^14,15^. Recent SAR satellites such as Sentinel-1 and ALOS-2 cover large ground swaths (respectively 250 and 350 km wide) while revisiting the same area on a regular interval (respectively 6 and 14 days). Interferometric SAR (InSAR) refers to the phase difference between two SAR images acquired over the same location. To generate an interferogram, both SAR images needs to be aligned accurately, and the contribution of the flat earth and topography removed^14,25^.

InSAR analysis is complicated by: (1) decorrelation noise introduced by a change in the satellite acquisition geometry and surface scattering properties^15^, and (2) atmospheric noise from the ionosphere and troposphere^25,26^.

We reduce the problem of decorrelation noise by applying advanced time-series InSAR processing using the Stanford Method of Persistent Scatterer (StaMPS)^14^. This method selects only those pixels that remain stable over time, decreasing the noise level. After time-series processing, we find interferograms with an average phase (scattering and thermal) noise of 25 degrees equivalent to an ~0.85 cm average precision for each interferograms. For unwrapping we use the iterative approach as proposed in Hussain *et al*.^27^. Prior to time-series processing using StaMPS, we perform the interferometric processing using the JPL InSAR Scientific Computing Environment (ISCE) software^28^ and use the SRTM DEM in our processing^29^.

The atmospheric noise can be split into ionospheric and tropospheric components^26^. The ionospheric noise is typically of a long spatial wavelength (>100 km) and, being a dispersive process, manifests more strongly in long wavelength SAR systems such as L-band^25^. The magnitude of the tropospheric InSAR noise is independent of the SAR wavelength, and becomes even more apparent for larger study areas (e.g., >20 km) and in the presence of topographic relief (>100’s m). It is mainly spatial and temporal variations of pressure, temperature, and relative humidity in the lower part of the troposphere which lead to a spatially variable tropospheric signal in InSAR^30^. Over our study area tropospheric topography-correlated noise is expected to be small as topography varies smoothly from -55 m to 15 m (SRTM WGS84 heights). We correct InSAR with GPS to account for residual long-wavelength errors such as orbit errors and residual atmospheric noise. By limiting to relatively small study areas, the ionosphere manifests as a long-wavelength signal which can generally be removed by setting a local reference area. For those study areas that exhibit strong ionospheric noise, contaminated SAR acquisitions are rejected. The tropospheric noise is further reduced through time-series processing, as noise gets averaged over time. After time-series InSAR processing, a map of average radar line-of-sight surface velocities is obtained along with associated uncertainties. The subsidence map follows from projecting the line-of-sight rates into to the vertical and referencing to local GPS stations while propagating full uncertainties. We therefore assume negligible horizontal motion for InSAR. The impact of this assumption is expected small as the horizontal unit vectors have a smaller contribution when projecting into the radar line-of-sight than the vertical and with horizontal rates that are on average an order of magnitude smaller than the vertical rate (see Table 2). Linear rates were computed for each GPS station and then used in the analysis. Atmopsheric InSAR noise becomes more apparent with distance from a reference point, reaching at 20 km distance uncertainties of up to 1 cm/yr. Common practice is to reference InSAR observations to GPS by fitting a plane and miniziming the residuals^14,27,31^. We found a simple planar fitting not to be sufficient to account for the variability of the ionospheric noise and had too few GPS stations to constrain a higher degree plane. Instead, we address this problem by utilizing only InSAR observations within a 20 km radius of a GPS station, allowing us to limit the impact of longer-walvength atmospheric InSAR noise. Given the local InSAR thermal and scattering noise, we average InSAR rates within a 500 m radius of the GPS site when referencing all InSAR observations within 20 km of that station. We require at least 50 InSAR observations (stable and well sampled) to justify using the station in the GPS reference frame tie-in. The associated uncertainty of the InSAR rate tied to the local GPS site follows by propagating the GPS and InSAR uncertainty, with the InSAR uncertainty estimated through bootstrapping the InSAR time-series while defining the GPS station location as a stable or reference point. By repeating this for all GPS stations, we obtain a local tied InSAR rate map with corresponding uncertainty for each station. Next, we compute the weighted average of the the locally tied rate maps and propagate the associated uncertainty to obtain the rate and uncertainty mapped tied to the complete GPS network. Locations in the vicinity of multiple GPS stations (where circles overlap) will have the smallest rate uncertainties. Given the precision and atmospheric noise of individual interferograms in combination with the ALOS acquisition sampling and uncertainty of the GPS vertical rates, we find at best an uncertainty of 3–4 mm/yr for the derived subsidence map.

### Data Availability

ALOS images and GPS rates are available for free from respectively the Alaska Satellite Facility (ASF, https://www.asf.alaska.edu/) and the Nevada Geodetic laboratory (http://geodesy.unr.edu/). ALOS dataset:^©^ JAXA/METI ALOS PALSAR L1.0 2007–2012. Accessed through ASF DAAC December 2016. ENVI files of the subsidence map with corresponding uncertainy map as generated in this study are included as supplemental material. Maps in Figs. 2, 4, 5 and 6 were created using ArcGIS® software by Esri. ArcGIS® and ArcMap™ are the intellectual property of Esri and are used herein under license. Copyright^©^ Esri. All rights reserved. For more information about Esri® software, please visit www.esri.com.

## Electronic supplementary material

Supplemental information

Dataset 1

Dataset 2
