# Supplementary material for: Spaceborne Synthetic Aperture Radar Survey of Subsidence in Hampton Roads, Virginia (USA)
Source: Sci Rep. 2017 Nov 7;7:14752. doi: 10.1038/s41598-017-15309-5 (PMC5677032; doi:10.1038/s41598-017-15309-5)
Supplement: Supplementary file 1 — Supplemental information [file 41598_2017_15309_MOESM1_ESM.pdf]

Supplemental material for:

Spaceborne Synthetic Aperture Radar Survey of Subsidence in Hampton Roads, Virginia (USA)

D.P.S. Bekaert[1]*, B.D. Hamlington[2], B. Buzzanga[2], C. E. Jones[1]

[1] *Jet Propulsion Laboratory, California Institute of Technology, CA, USA.

[2] Center for Coastal Physical Oceanography, Old Dominion University, Norfolk, USA.

*Corresponding author: david.bekaert@jpl.nasa.gov

Supplementary Data 1

Vup.zip contains the data ENVI file and header of vertical subsidence rate over Hampton Roads area [mm/yr] referenced to GPS (NA12 frame). Generated subsidence map combines ALOS InSAR data with and local vertical GPS rates. Corresponding uncertainties are contained in Supplementary Data 2.

Supplementary Data 2

Vup_std.zip contains the ENVI file and header of vertical subsidence rate uncertainty (1-sgima) over Hampton Roads area [mm/yr] referenced to GPS (NA12 frame). Corresponding subsidence rates are contained in Supplementary Data 1.
